# Using interval unions to solve linear systems of equations with uncertainties

**DOI:** 10.1007/s10543-017-0657-x

**Published:** 2017-04-22

**Authors:** Tiago Montanher, Ferenc Domes, Hermann Schichl, Arnold Neumaier

**Affiliations:** Faculty of Mathematics, University of Vienna, Oskar-Morgenstern-Platz 1, 1090 Vienna, Austria

**Keywords:** Interval union arithmetic, Interval union linear systems, Interval union Gauss–Seidel, Rigorous numerical linear algebra, 65F10, 65G20, 65G30, 65G40

## Abstract

An interval union is a finite set of closed and disjoint intervals. In this paper we introduce the interval union Gauss–Seidel procedure to rigorously enclose the solution set of linear systems with uncertainties given by intervals or interval unions. We also present the interval union midpoint and Gauss–Jordan preconditioners. The Gauss–Jordan preconditioner is used in a mixed strategy to improve the quality and efficiency of the algorithm. Numerical experiments on interval linear systems generated at random show the capabilities of our approach.

## Introduction

In traditional interval arithmetic, division by an interval containing zero overestimates the range when the latter is disconnected. Treating this using complements of intervals (see e.g, [23]) only postpones the problem a little, while interval union arithmetic, introduced by [24] as arithmetic on finite ordered sets of disjoint closed, possibly unbounded intervals, allow a mathematically and computationally natural approach to this problem. Indeed, the collection of interval unions (treated as closed sets in the obvious way) is closed under set-theoretic addition, subtraction, multiplication, division (after adding end points in case of an unbounded divisor), and all continuous elementary operations.

Many theoretical results from interval analysis remain valid for interval unions. For example, elementary operations and standard functions are inclusion isotone and the fundamental theorem of interval analysis also generalizes to interval unions. On the other hand, properties based on convexity (like the interval mean value theorem) do not apply to interval unions.

In this paper we study the rigorous solution of interval union linear systems of equations (IULS). We denote interval unions and vectors of interval unions by bold calligraphic letters (such as $\fancyscript{a}$, $\fancyscript{x}$), while matrices of interval unions are denoted by capital bold calligraphic letters (e.g., $\mathcal {A}$, $\mathcal {B}$). Let $\mathcal {A}$ and $\fancyscript{b}$ be a matrix and a vector with interval union entries respectively. If $\fancyscript{x}$ is a given initial interval union vector, we are interested in finding an enclosure of the solution set for the family of equations

1 $$\begin{aligned} A x = b,\quad (A \in \mathcal {A}, b \in \fancyscript{b}, x \in \fancyscript{x}). \end{aligned}$$

This problem has several applications in rigorous numerical analysis. Since interval linear systems are embedded into the interval union framework, any algorithm that relies on the rigorous solution of interval linear systems can benefit from the methods discussed in this paper. For example, constraint propagation methods [5] and the interval Newton operator [20, 21] can be significantly improved with the use of interval union techniques. Moreover, interval union linear systems of equations can be used to define an interval union branch and bound framework for rigorous global optimization. This application will be detailed in a future work.

*Related work*: A closely related concept is that of multi-intervals, introduced independently by Yakovlev [28] and Telerman (see Telerman et al. [26]). According to [27], they are defined as a union of closed intervals that are not necessarily disjoint, making them slightly more general from the interval unions of the present paper.

Multi-interval arithmetic is (a not separately accessible) part of the publicly available software *Unicalc* [1, 22] for solving constraint satisfaction problems and nonlinear systems of equations. Another implementation of multi-intervals is described in [25]. Parallel algorithms for interval and multi-interval arithmetic are the subject of [17]. Kreinovich et al. [18] use multi-intervals to study the existence of algorithms to solve algebraic systems. No systematic performance evaluation seems to be known. Multi-intervals were also applied to the analysis of analog circuits [7], to the modeling of financial models under partial uncertainty [19], and to bit-width optimization [2].

Another variant of interval unions are the discontinuous intervals by Hyvönen [11], applied in [12, 13] to simple constraint satisfaction problems and spreadsheet computations. They are disjoint unions of closed, half-open, or open intervals. In our opinion, the extra bookkeeping effort to distinguished between closed and open endpoints is not warranted in most applications.

*Content*: We organized this paper as follows: Sect. 2 summarizes the fundamentals of the interval union arithmetic. In Sect. 3, we define interval union matrices, vectors and linear systems of equations.

In Sect. 4, we introduce two forms of the interval union Gauss–Seidel procedure to solve (1): the partial form and the complete form. In the partial form, we update only the variable corresponding to the main diagonal entry of *A* at each iteration. In the complete form, we update all variables in each row.

Preconditioner heuristics are the subject of Sect. 5. Interval algorithms usually precondition the initial interval linear system to improve the quality of the solution. We extend the idea of preconditioning to interval unions and study two different preconditioning heuristics. The first one is the midpoint method: it takes the inverse of the midpoint of the hull matrix of the system $\mathcal {A}$ as the preconditioner. The second one is the Gauss–Jordan preconditioner which is based on the Gauss–Jordan elimination as discussed in [6].

Since solving large systems—due to the cost of the matrix multiplication required in the preconditioning heuristics—becomes intractable, we propose a mixed strategy that combines the original system with its preconditioned form.

Section 6 presents results of our numerical experiments. We consider randomly generated interval linear systems in order to compare traditional interval methods with the our new approach. We take linear systems with $n \in \{2, 3, 5, 10, 15, 20, 30, 50\}$ where entries of $\mathbf{A}$, $\mathbf{b}$ and $\mathbf{x}$ have radius $r \in \{0.1, 0.2,\ldots , 2.9, 3.0\}$.

The experiment shows that interval union methods produce better enclosures than their interval counterparts. The interval union Gauss–Seidel procedure with and without preconditioners produce enclosures up to $25\%$ sharper than those obtained by interval methods. Moreover, there are no significant differences between the execution time of intervals and interval union methods.

*Notation*: We denote the vector space of all $m \times n$ matrices *A* with real entries $A_{ik}$ ($i=1,\ldots ,m,~k=1,\ldots ,n$) by $\mathbb {R}^{m\times n}$. The vector space of all column vectors *v* of length *n* and entries $v_{i}$ is denoted by $\mathbb {R}^n=\mathbb {R}^{n\times 1}$.

The *n*-dimensional identity matrix is given by $I$. We denote the set of induces $1,\ldots , N$ by 1 : *N* and write $A_{i:}$ and $A_{:j}$ to denote the *i*-th row and *j*-th column of the matrix *A* respectively.

We assume that the reader is familiar with basic interval arithmetic. A comprehensive approach to this subject is given by [21]. For the interval arithmetic notation, we mostly follow [16]. Let $\underline{a}, \overline{a} \in \mathbb {R}$ with $\underline{a} \le \overline{a}$ then $\mathbf{a}=[\underline{a}, \overline{a}]$ denotes an interval with $\inf (\mathbf{a}) := \min (\mathbf{a}) := \underline{a}$ and $\sup (\mathbf{a}) := \max (\mathbf{a}) := \overline{a}$. The set of nonempty compact real intervals is given by

$$\begin{aligned} \mathbb {I}\mathbb {R}:= \{[\underline{a}, \overline{a}] \mid \underline{a} \le \overline{a},~ \underline{a}, \overline{a} \in \mathbb {R}\}. \end{aligned}$$

We will allow the extremes of the intervals to assume the ideal points $-\infty$ and $\infty$, and define $\overline{\mathbb {IR}}$ as the set of closed real intervals and write

$$\begin{aligned} \overline{\mathbb {IR}}:= \left\{ [\underline{a}, \overline{a}] \cap \mathbb {R}\mid \underline{a} \le \overline{a}, ~ \underline{a}, \overline{a} \in \mathbb {R}\cup \{-\infty , \infty \} \right\} , \end{aligned}$$

The width of the interval $\mathbf{a}\in \overline{\mathbb {IR}}$ is given by $\hbox {wid}(\mathbf{a}):=\overline{a}-\underline{a}$, its magnitude by $|~\!\mathbf{a}|~\!:= \max (|\underline{a}|, |\overline{a}|)$ and its mignitude by

$$\begin{aligned} \left\langle \mathbf{a} \right\rangle :=\left\{ \begin{array}{ll} \min (|\underline{a}|, |\overline{a}|) &{}\quad \text{ if } 0 \notin [\underline{a}, \overline{a}] ,\\ 0 &{}\quad \text{ otherwise }. \end{array}\right. \end{aligned}$$

The midpoint of $\mathbf{a}\in \mathbb {IR}$ is $\check{\mathbf{a}}:=\hbox {mid}(\mathbf{a}):=( \underline{a} + \overline{a})/2$ and the radius of $\mathbf{a}\in \overline{\mathbb {IR}}$ is $\hat{\mathbf{a}}:= \hbox {rad}(\mathbf{a}):=( \underline{a}-\overline{a})/2$. An interval is called degenerate if $\hbox {wid}(\mathbf{a}) = 0$.

For any set $S \subseteq \mathbb {R}$, the smallest interval containing *S* is called the interval hull of *S* and denoted by . The notions of elementary operations between intervals and inclusion properties are the same as presented in [21]. If $\mathbf{a},\mathbf{b}\in \mathbb {IR}$ then the extended division is defined as follows (see e.g, [23])

2 $$\begin{aligned} \mathbf{a}/\mathbf{b}:= \left\{ \begin{array}{ll} \mathbf{a}* [1/\overline{b},1/\underline{b}] &{}\quad \text{ if } 0\notin \mathbf{b}, \\ {(}{-}\infty ,+\infty ) &{}\quad \text{ if } 0\in \mathbf{a}\wedge 0\in \mathbf{b}, \\ {[}\overline{a}/\underline{b},+\infty ) &{}\quad \text{ if } \overline{a}<0 \wedge \underline{b}< \overline{b} = 0,\\ {(}{-}\infty ,\overline{a}/\overline{b}] \cup {[}\overline{a}/\underline{b},+\infty ) &{}\quad \text{ if } \overline{a}<0 \wedge \underline{b}< 0< \overline{b},\\ {(}{-}\infty ,\overline{a}/\overline{b}] &{}\quad \text{ if } \overline{a}<0 \wedge 0 = \underline{b}< \overline{b},\\ {(}{-}\infty ,\underline{a}/\underline{b}] &{}\quad \text{ if } 0<\underline{a} \wedge \underline{b}< \overline{b} = 0,\\ {(}{-}\infty ,\underline{a}/\underline{b}] \cup {[}\underline{a}/\overline{b},+\infty ) &{}\quad \text{ if } 0<\underline{a} \wedge \underline{b}< 0< \overline{b},\\ {[}\underline{a}/\overline{b},+\infty ) &{}\quad \text{ if } 0<\underline{a} \wedge 0 = \underline{b} < \overline{b},\\ \emptyset &{}\quad \text{ if } 0\notin \mathbf{a}\wedge \underline{b}=\overline{b}=0. \end{array}\right. \end{aligned}$$

An interval vector $\mathbf{x}=[\underline{x},\overline{x}]$ is the Cartesian product of the closed real intervals $\mathbf{x}_i:=[\underline{x}_i, \overline{x}_i] \in \overline{\mathbb {IR}}$. We denote the set of all interval vectors of dimension *n* by $\overline{\mathbb {IR}}^{n}$. We denote interval matrices by capital bold letters ($\mathbf{A}$, $\mathbf{B}$, ...) and the set of all $m \times n$ interval matrices is given by $\overline{\mathbb {IR}}^{m \times n}$.

For some applications, the interval subtraction may over-estimate the range of the real computation. For example, since $-\mathbf{a}:= 0 - \mathbf{a}= [-\sup (\mathbf{a}), -\inf (\mathbf{a})]$ then

$$\begin{aligned} \mathbf{b}:= \mathbf{a}+ (-\mathbf{a}) = [\inf (\mathbf{a}) - \sup (\mathbf{a}), \sup (\mathbf{a}) - \inf (\mathbf{a})] \end{aligned}$$

and $\mathbf{b}= [0, 0]$ only if $\inf (\mathbf{a}) = \sup (\mathbf{a})$. In order to cope with this situation we also define inner subtraction for intervals. If $\mathbf{a},\mathbf{b}\in \overline{\mathbb {IR}}$ then

3 $$\begin{aligned} \mathbf{a}\ominus \mathbf{b}:= \left\{ \begin{array}{ll} {[} \inf (\mathbf{a}) - \inf (\mathbf{b}), \sup (\mathbf{a}) - \sup (\mathbf{b}) ] &{}\quad \text{ if } \hbox {wid}(\mathbf{a}) \ge \hbox {wid}(\mathbf{b})\\ {[} \sup (\mathbf{a}) - \sup (\mathbf{b}), \inf (\mathbf{a}) - \inf (\mathbf{b})] &{}\quad \text{ otherwise. } \\ \end{array}\right. \end{aligned}$$

For a comprehensive review of inner operations, see [3].

## Interval unions

This section introduces the basics of interval unions. For more details on the topics covered in this section see [24].

### Definition 1

An interval union $\fancyscript{u}$ of length $l(\fancyscript{u}):=k$ is a finite set of *k* intervals of form

$$\begin{aligned} \fancyscript{u}:= (\mathbf{u}_1, \dots , \mathbf{u}_k)~~\text {with}~~ \begin{array}{ll} \mathbf{u}_i \in \overline{\mathbb {IR}}&{}\quad \forall ~ i = 1, \dots , k,\\ \overline{\mathbf{u}}_{i} < \underline{\mathbf{u}}_{i+1} &{}\quad \forall ~ i = 1, \dots , k-1. \end{array} \end{aligned}$$

We denote by ${\mathcal {U}}_k$ the set of all interval unions of length $\le k$. The set of all interval unions is then ${\mathcal {U}}:=\bigcup _{k \ge 0} {\mathcal {U}}_k$ where we define ${\mathcal {U}}_0 := \emptyset$.

If $\fancyscript{u}\in {\mathcal {U}}$ is an interval union with $l(\fancyscript{u}) = k$ then for any $x \in \mathbb {R}$ we say

$$\begin{aligned} x \in \fancyscript{u}~~~\Leftrightarrow ~~~\text{ there } \text{ exists } \text{ a } 1\le i\le k \text{ such } \text{ that } x \in \mathbf{u}_i. \end{aligned}$$

The relation above extends naturally for intervals and another interval unions, so that if $\fancyscript{v}$ is an interval union then

$$\begin{aligned} \fancyscript{v}\subseteq \fancyscript{u}~~~\Leftrightarrow ~~~\hbox { for all } {{\varvec{v}}}\in \fancyscript{v}\hbox { there exists a }1\le i\le k \hbox { such that } {{\varvec{v}}}\subseteq {\mathbf{u}}_i. \end{aligned}$$

Let *S* be a set of *k* intervals with $k < \infty$. The smallest interval union with respect to inclusion that satisfies $\mathbf{a}\subseteq \fancyscript{u}$ for all $\mathbf{a}\in S$ is called the union creator ${\mathcal {U}}(S)$ of *S*. Formally we have

4 $$\begin{aligned} {\mathcal {U}}(S) := \{ u \in \overline{\mathbb {R}}\mid u \in \cup _{i = 1}^{k} S_{i} \}. \end{aligned}$$

Clearly, ${\mathcal {U}}(S) \in {\mathcal {U}}_{k}$, ${\mathcal {U}}({\mathcal {U}}(S)) = {\mathcal {U}}(S)$ and $S_{1} \subseteq S_{2}$ implies ${\mathcal {U}}(S_{1}) \subseteq {\mathcal {U}}(S_{2})$. The interval hull of a union $\fancyscript{u}\in {\mathcal {U}}$ is denoted by .

Let $\fancyscript{u}\in {\mathcal {U}}_{k} \setminus \{\emptyset \}$. The magnitude and mignitude of $\fancyscript{u}$ are given by

$$\begin{aligned} |\fancyscript{u}|:= \max (|\mathbf{u}_{1}|,\ldots , |\mathbf{u}_{k}|) = \max (|\underline{\mathbf{u}}_{1}|, |\overline{\mathbf{u}}_{k}|) \end{aligned}$$

and

$$\begin{aligned} \left\langle \fancyscript{u} \right\rangle := \min (\left\langle \mathbf{u}_{1} \right\rangle ,\ldots , \left\langle \mathbf{u}_{k} \right\rangle ). \end{aligned}$$

The maximum, minimum and maximum width of the non-empty interval union $\fancyscript{u}$ are defined by

$$\begin{aligned} \max (\fancyscript{u}):= \overline{\mathbf{u}}_{k}, \quad \min (\fancyscript{u}):= \underline{\mathbf{u}}_{1} \end{aligned}$$

and

$$\begin{aligned} \max \hbox {wid}(\fancyscript{u}):= \max (\hbox {wid}(\mathbf{u}_{1}),\ldots , \hbox {wid}(\mathbf{u}_{k})). \end{aligned}$$

The projection of the point $x \in \mathbb {R}$ into the interval union $\fancyscript{u}\in {\mathcal {U}}_{k}$ is given by

$$\begin{aligned} \hbox {proj}(x, \fancyscript{u}) := \left\{ \begin{array}{l@{\quad }ll} x &{} \text{ if } &{} x \in \fancyscript{u}\\ \overline{\mathbf{u}}_{i} &{} \text{ if } &{} x \in {]} \overline{\mathbf{u}}_{i}, \underline{\mathbf{u}}_{i+1}{[} \text{ and } x - \overline{\mathbf{u}}_{i}< \underline{\mathbf{u}}_{i+1} - x,\\ \underline{\mathbf{u}}_{i+1} &{} \text{ if } &{} x \in {]}\overline{\mathbf{u}}_{i}, \underline{\mathbf{u}}_{i+1}{[} \text{ and } x - \overline{\mathbf{u}}_{i} \ge \underline{\mathbf{u}}_{i+1} - x,\\ \overline{\mathbf{u}}_{k} &{} \text{ if } &{} x > \overline{\mathbf{u}}_{k},\\ \underline{\mathbf{u}}_{1} &{} \text{ if } &{} x < \underline{\mathbf{u}}_{1}.\\ \end{array}\right. \end{aligned}$$

### Definition 2

Let $\mathbf{x}\in \mathbb {IR}$ be an interval, $\fancyscript{u}:=(\mathbf{u}_1, \dots , \mathbf{u}_k)$ and $\fancyscript{s}:=(\mathbf{s}_1, \dots \mathbf{s}_t)$ interval unions and let $\circ _\bullet \in \{+,-,/,*, \ominus \}$ be an elementary interval operation with the division operator given by (2) and the inner subtraction by (3).

(i) The elementary interval union operation $\circ _\star : {\mathcal {U}}\times \overline{\mathbb {IR}}\rightarrow {\mathcal {U}}$ is given by
  $$\begin{aligned} \fancyscript{u}\circ _\star \mathbf{x}:={\mathcal {U}}(\left\{ \mathbf{u}_1 \circ _\bullet \mathbf{x}, \dots , \mathbf{u}_k \circ _\bullet \mathbf{x}\right\} ). \end{aligned}$$
(ii) The elementary interval union operation $\circ _\star : {\mathcal {U}}\times {\mathcal {U}}\rightarrow {\mathcal {U}}$ is given by
  $$\begin{aligned} \fancyscript{u}\circ \fancyscript{s}:={\mathcal {U}}(\{\fancyscript{u}\circ _\star \mathbf{s}_1, \dots , \fancyscript{u}\circ _\star \mathbf{s}_t\}). \end{aligned}$$

The following result gives basic properties of interval union arithmetic, see [24].

### Proposition 1

Let $\fancyscript{u}, \fancyscript{v}$ and $\fancyscript{s}$ be interval unions. Then for $\circ \in \{+,-,/,*\}$,

5 $$\begin{aligned}&\fancyscript{u}\subseteq \fancyscript{u}',~ \fancyscript{s}\subseteq \fancyscript{s}' \Longrightarrow \fancyscript{u}\circ \fancyscript{s}\subseteq \fancyscript{u}' \circ \fancyscript{s}' \end{aligned}$$

6 $$\begin{aligned}&\fancyscript{u}(\fancyscript{v}\pm \fancyscript{s}) \subseteq \fancyscript{u}\fancyscript{v}\pm \fancyscript{u}\fancyscript{s}. \end{aligned}$$

7 $$\begin{aligned}&a(\fancyscript{u}+ \fancyscript{v}) = a\fancyscript{u}+ a\fancyscript{v}~~~\text { for } a \in \mathbb {R}, \end{aligned}$$

## Interval union vectors, matrices and linear systems

### Definition 3

An $m \times n$ interval union matrix is a rectangular array of interval unions with *m* rows and *n* columns. We denote interval union matrices by capital bold calligraphic letters ($\mathcal {A}$, $\mathcal {B}$, ...) and the (*i*, *j*)—element of the interval union matrix $\mathcal {A}$ is given by $\mathcal {A}_{ij}$. The set of $m \times n$ interval union matrices is given by ${\mathcal {U}}^{m\times n}$. In a similar way, $n \times 1$ interval union matrices are called interval union vectors. We denote interval union vectors by bold calligraphic letters ($\varvec{u}$, $\fancyscript{x}$, ...) and the set of all *n*-dimensional interval union vectors is given by ${\mathcal {U}}^{n}$. We denote the set of *n*-dimensional vectors $\varvec{u}$ satisfying $l(\varvec{u}_{i}) = k_{i}$ by ${\mathcal {U}}_{k_{1},\ldots ,k_{n}}^{n}$.

Given a set of interval vectors $\{\varvec{u}_{1},\ldots , \varvec{u}_{p}\}$, the union creator vector is denoted by $\fancyscript{v}:= {\mathcal {U}}(\{\varvec{u}_{1},\ldots , \varvec{u}_{p}\})$ where the union creator ${\mathcal {U}}$ defined in (4) is applied component-wise. Let $\fancyscript{u}$ be an *n*-dimensional interval union vector satisfying $l(\fancyscript{u}_{i}) = k_{i}$ and $p = \prod _{i = 1}^{n} k_{i}$. If we denote the Cartesian product between two interval unions by $\times$ then the mapping $\mathcal {S}: {\mathcal {U}}_{k_{1},\ldots ,k_{n}}^{n} \rightarrow (\mathbb {IR}^{n})^{p}$ given by

$$\begin{aligned} \mathcal {S}(\fancyscript{v}) := \fancyscript{v}_{1} \times \fancyscript{v}_{2} \times \cdots \times \fancyscript{v}_{n} \end{aligned}$$

splits the interval union $\varvec{u}$ into a set of *p* disjoint interval vectors. Notice that interval union vectors can be used to represent *p* disjoint interval vectors storing only $\sum _{i = 1}^{n} k_{i}$ elements. This is a clear advantage over traditional interval arithmetic, especially when *n* is large. The mapping $\mathcal {S}$ and the definition of union creator can be naturally extended to matrices.

Interval union matrices and vectors follow the usual definition of arithmetic operations. Formally, if $\mathcal {A}, \mathcal {B}\in {\mathcal {U}}^{m \times n}$ and $\mathcal {C}\in {\mathcal {U}}^{n \times p}$ then

8 $$\begin{aligned} (\mathcal {A}\pm \mathcal {B})_{ij} := \mathcal {A}_{ij} \pm \mathcal {B}_{ij} \end{aligned}$$

and

9 $$\begin{aligned} (\mathcal {A}\mathcal {C})_{ij} := \sum _{k = 1}^{n} \mathcal {A}_{ik} \mathcal {C}_{kj}. \end{aligned}$$

### Proposition 2

Let $\mathcal {A}, \mathcal {A}', \mathcal {B}, \mathcal {B}' \in {\mathcal {U}}^{m \times n}$ and $\mathcal {C}, \mathcal {C}' \in {\mathcal {U}}^{n \times p}$. Then

$$\begin{aligned}&\mathcal {A}' \subseteq \mathcal {A},~ \mathcal {B}' \subseteq \mathcal {B}~~~\Rightarrow ~~~\mathcal {A}' \pm \mathcal {B}' \subseteq \mathcal {A}\pm \mathcal {B},\\&\mathcal {A}' \subseteq \mathcal {A},~ \mathcal {C}' \subseteq \mathcal {C}~~~\Rightarrow ~~~\mathcal {A}'\mathcal {C}' \subseteq \mathcal {A}\mathcal {C},\\&\mathcal {A}(\mathcal {C}+ \mathcal {C}') \subseteq \mathcal {A}\mathcal {C}+ \mathcal {A}\mathcal {C}',\\&A(\mathcal {C}+ \mathcal {C}') = A \mathcal {C}+ A \mathcal {C}' ~~~\text { for } A \in \mathbb {R}^{m \times n},\\&(\mathcal {A}+ \mathcal {A}')C = \mathcal {A}C + \mathcal {A}' C ~~~\text { for } C \in \mathbb {R}^{n \times p}. \end{aligned}$$

### Proof

Follows from Relations (5)–(7) applied to Definitions (8) and (9). $\square$

An interval union linear system of equations (ILLS) with coefficients $\mathcal {A}\in {\mathcal {U}}^{n \times n}$ and $\fancyscript{b}\in {\mathcal {U}}^{n}$ is the family of linear equations

10 $$\begin{aligned} A x = b ~~~ (A \in \mathcal {A}, b \in \fancyscript{b}). \end{aligned}$$

This paper deals only with square systems though the generalization to systems of form $m \times n$ is straightforward. The solution set of (10) is defined by

11 $$\begin{aligned} \varSigma (\mathcal {A}, \fancyscript{b}) := \{x \in \mathbb {R}^{n} \mid A x = b \text { for some } A \in \mathcal {A}, b \in \fancyscript{b}\}. \end{aligned}$$

As in the interval case, (11) can be a non-convex or disconnected set. Let $\fancyscript{x}_{0} \in {\mathcal {U}}^{n}$ be an interval union vector. The truncated solution set of (10) is

12 $$\begin{aligned} \varSigma (\mathcal {A}, \fancyscript{b})\cap \fancyscript{x}_{0} := \{x \in \fancyscript{x}_{0} \mid A x = b \text { for some } A \in \mathcal {A}, b \in \fancyscript{b}\}. \end{aligned}$$

The following proposition states that (11) is identical to the union of solution sets from the interval components of $\mathcal {A}$ and $\fancyscript{b}$.

### Proposition 3

Let $\mathcal {A}\in {\mathcal {U}}^{n \times n}$ and $\fancyscript{b}\in {\mathcal {U}}^{n}$. Then

$$\begin{aligned} \bigcup _{\begin{array}{c} \mathbf{A}_{i} \in \mathcal {S}(\mathcal {A})\\ \mathbf{b}_{j} \in \mathcal {S}(\fancyscript{b}) \end{array}} \varSigma (\mathbf{A}_{i}, \mathbf{b}_{j}) \equiv \varSigma (\mathcal {A}, \fancyscript{b}). \end{aligned}$$

### Proof

Let $$x \in \bigcup _{\begin{array}{c} \mathbf{A}_{i} \in \mathcal {S}(\mathcal {A})\\ \mathbf{b}_{j} \in \mathcal {S}(\fancyscript{b}) \end{array}} \varSigma (\mathbf{A}_{i}, \mathbf{b}_{j})$$. Then for some *i* and *j* there exist $A \in \mathbf{A}_{i}$ and $b \in \mathbf{b}_{j}$ such that $A x = b$. Since $\mathbf{A}_{i} \in \mathcal {A}$ and $\mathbf{b}_{j} \in \fancyscript{b}_{j}$ follows that $x \in \varSigma (\mathcal {A}, \fancyscript{b})$. Conversely, if $x \in \varSigma (\mathcal {A}, \fancyscript{b})$ then $A x = b$ for some $A \in \mathcal {A}$ and $b \in \fancyscript{b}$. The result follows from the definition of $\mathcal {S}(\mathcal {A})$ and $\mathcal {S}(\fancyscript{b})$. $\square$

Let $\mathbf{A}$ and $\mathbf{b}$ be an interval matrix and vector respectively. The problem of finding  and  is known to be *NP*–hard (see, e.g., [8, 18]). Therefore, Proposition 3 implies that finding ${\mathcal {U}}(\varSigma (\mathcal {A}, \fancyscript{b}))$ and ${\mathcal {U}}(\varSigma (\mathcal {A}, \fancyscript{b})\cap \fancyscript{x}_{0})$ are also *NP*–hard problems. This paper focuses on algorithms to enclose ${\mathcal {U}}(\varSigma (\mathcal {A}, \fancyscript{b})\cap \fancyscript{x}_{0})$. Formally, we are interested in finding nontrivial vectors $\fancyscript{y}$ (i.e $\fancyscript{y}\ne \fancyscript{x}_{0}$) satisfying

$$\begin{aligned} {\mathcal {U}}(\varSigma (\mathcal {A}, \fancyscript{b})\cap \fancyscript{x}_{0}) \subseteq \fancyscript{x}_{0} \subseteq \fancyscript{y}. \end{aligned}$$

Proposition 3 gives a natural approach to this problem. It consists in the application of the interval Gauss–Seidel procedure described in [9, 14, 21] to each system obtained by splitting $\mathcal {A}$ and $\fancyscript{b}$.

Let $$p = \prod _{\begin{array}{c} i = 1:N, \\ j = 1:N \end{array}} l(\mathcal {A}_{ij})$$, $q = \prod _{i = 1:N} l(\fancyscript{b}_{i})$ and $r = \prod _{i = 1:N} l(\fancyscript{x}_{i})$. The method proposed above requires the solution of $p\cdot q\cdot r$ interval linear systems of equations and does not take the structure of the interval union matrix and vector into account. The next section presents extensions of the Gauss–Seidel procedure to interval unions. We show that even in problems where $\mathcal {A}\in {\mathcal {U}}^{n \times n}_{1}$ and $\fancyscript{b}\in {\mathcal {U}}^{n}_{1}$, interval union algorithms give better results than their interval counterparts.

The interval union matrix $\mathcal {A}\in {\mathcal {U}}^{n \times n}$ is said to be regular if every real matrix $A \in \mathcal {A}$ is nonsingular. The interval union inverse of a regular matrix $\mathcal {A}$ is given by

$$\begin{aligned} \mathcal {A}^{-1} := {\mathcal {U}}\left( \left\{ A^{-1} \mid A \in \mathcal {A}\right\} \right) . \end{aligned}$$

### Proposition 4

Let $\mathcal {A}\in {\mathcal {U}}^{n \times n}$ be a regular matrix and $\fancyscript{b}\in {\mathcal {U}}^{n \times 1}$. Then

$$\begin{aligned} \varSigma (\mathcal {A}, \fancyscript{b}) \subseteq \mathcal {A}^{-1}\fancyscript{b}:= {\mathcal {U}}(\{ x \in \mathbb {R}^{n} \mid x = A^{-1}b ~\text { for some } A \in \mathcal {A}, b \in \fancyscript{b}\}). \end{aligned}$$

### Proof

Let $x \in \varSigma (\mathcal {A}, \fancyscript{b})$. Then there are $A \in \mathcal {A}$ and $b \in \fancyscript{b}$ such that $A x = b$. Since $\mathcal {A}$ is regular, $A^{-1}$ is well defined and therefore $x \in \mathcal {A}^{-1}\fancyscript{b}$. $\square$

## The interval union Gauss–Seidel method

Let $\mathcal {A}\in {\mathcal {U}}^{n \times n}$, $\fancyscript{b}\in {\mathcal {U}}^{n}$ and $\fancyscript{x}_{0} \in {\mathcal {U}}^{n}$. In this section we introduce the interval union Gauss–Seidel procedure to rigorously enclose the solution set of

$$\begin{aligned} A x = b \quad (A \in \mathcal {A}, b \in \fancyscript{b}, x \in \fancyscript{x}_{0}). \end{aligned}$$

We first discuss the univariate interval union Gauss–Seidel operator and show its properties using the definitions and results from [21].

For higher dimensions, we present two versions of the Gauss–Seidel procedure. In the first version, called the partial form, we update only the variable corresponding to $\mathcal {A}_{ii}$ in the *i*th row. In the second, named complete, we consider all variables at each iteration.

### Interval union Gauss–Seidel operator

Let $\fancyscript{a}, \fancyscript{b}, \fancyscript{x}\in {\mathcal {U}}$. The interval union linear system in this case reduces to

$$\begin{aligned} a x = b ~~~ (a \in \fancyscript{a}, b \in \fancyscript{b}, x \in \fancyscript{x}). \end{aligned}$$

As in the Definition (12), the truncated solution set is given by

13 $$\begin{aligned} \varSigma (\fancyscript{a}, \fancyscript{b})\cap \fancyscript{x}:= \{x \in \fancyscript{x}\mid a x = b \text { for some } a \in \fancyscript{a}, b \in \fancyscript{b}\}. \end{aligned}$$

The univariate interval union Gauss–Seidel operator is defined by

14 $$\begin{aligned} \varGamma (\fancyscript{a}, \fancyscript{b}, \fancyscript{x}) := {\mathcal {U}}(\{x \in \fancyscript{x}\mid a x = b \text { for some } a \in \fancyscript{a}, b \in \fancyscript{b}\}). \end{aligned}$$

#### Proposition 5

Let $$\fancyscript{a}, \fancyscript{b}, \fancyscript{x}\in {\mathcal {U}}$$ then

15 $$\begin{aligned}&\varGamma (\fancyscript{a}, \fancyscript{b}, \fancyscript{x}) = \frac{\fancyscript{b}}{\fancyscript{a}} \cap \fancyscript{x}, \end{aligned}$$

16 $$\begin{aligned}&\varSigma (\fancyscript{a}, \fancyscript{b})\cap \fancyscript{x}\equiv \varGamma (\fancyscript{a}, \fancyscript{b}, \fancyscript{x}) \subseteq \fancyscript{x}, \end{aligned}$$

17 $$\begin{aligned}&\varGamma (\fancyscript{a}, \fancyscript{b}, \fancyscript{x}) \equiv \varnothing ~~~\Rightarrow ~~~\varSigma (\fancyscript{a}, \fancyscript{b})\cap \fancyscript{x}\equiv \varnothing , \end{aligned}$$

18 $$\begin{aligned}&0 \notin \fancyscript{b}- \fancyscript{a}\fancyscript{x}~~~\Rightarrow ~~~\varSigma (\fancyscript{a}, \fancyscript{b})\cap \fancyscript{x}\equiv \varnothing , \end{aligned}$$

19 $$\begin{aligned}&0 \in \fancyscript{a},~ 0 \in \fancyscript{b}~~~\Rightarrow ~~~\varSigma (\fancyscript{a}, \fancyscript{b})\cap \fancyscript{x}\equiv \fancyscript{x}, \end{aligned}$$

20 $$\begin{aligned}&\fancyscript{a}' \subseteq \fancyscript{a},~ \fancyscript{b}' \subseteq \fancyscript{b},~ \fancyscript{x}' \subseteq \fancyscript{x}~~~\Rightarrow ~~~\varGamma (\fancyscript{a}', \fancyscript{b}', \fancyscript{x}') \subseteq \varGamma (\fancyscript{a}, \fancyscript{b}, \fancyscript{x}). \end{aligned}$$

#### Proof

From Definition 2, we have

$$\begin{aligned} \frac{\fancyscript{b}}{\fancyscript{a}} = {\mathcal {U}}(\{ x \in \mathbb {R}\mid \tilde{a} x = \tilde{b} \text { for some } \tilde{a} \in \fancyscript{a}, \tilde{b} \in \fancyscript{b}\}) \end{aligned}$$

and (15) follows from taking the intersection with $\fancyscript{x}$. To prove (16) note that Definitions (13) and (14) imply that $\varSigma (\fancyscript{a}, \fancyscript{b})\cap \fancyscript{x}\subseteq \varGamma (\fancyscript{a}, \fancyscript{b}, \fancyscript{x})$. Conversely, if $x \in \varGamma (\fancyscript{a}, \fancyscript{b}, \fancyscript{x})$ then Definition (4) implies that *x* is contained in some component of $\{x \in \fancyscript{x}\mid a x = b \text { for some } a \in \fancyscript{a}, b \in \fancyscript{b}\}$ and therefore $\varGamma (\fancyscript{a}, \fancyscript{b}, \fancyscript{x}) \subseteq \varSigma (\fancyscript{a}, \fancyscript{b})\cap \fancyscript{x}$. Relation (17) follows immediately from (16). If $0 \notin \fancyscript{b}- \fancyscript{a}\fancyscript{x}$ then there is no $a \in \fancyscript{a}$, $b \in \fancyscript{b}$ and $x \in \fancyscript{x}$ such that $ax = b$. Therefore $\{x \in \fancyscript{x}\mid a x = b\text { for some } a \in \fancyscript{a}, b \in \fancyscript{b}\}$ is empty and Relation (18) holds. Relations (19) and (20) follow immediately from the extended division in Definition (2) and the inclusion property respectively. $\square$

Let $\mathcal {A}\in {\mathcal {U}}^{n \times n}$, $\fancyscript{b}\in {\mathcal {U}}^{n}$, $A \in \mathcal {A}$ and $b \in \fancyscript{b}$. If $A_{ii} \ne 0$ and $\tilde{x} \in \fancyscript{x}$ is an approximation of the solution of $A x = b$ then the Gauss–Seidel iteration is given by

$$\begin{aligned} \tilde{x}_{i}' := \frac{b_{i} - \sum _{j = 1}^{i-1} A_{ij}\tilde{x}_{j}' - \sum _{j = i+1}^{n} A_{ij}\tilde{x}_{j}}{A_{ii}}. \end{aligned}$$

Since all elementary operations are inclusion isotone we have

21 $$\begin{aligned} \tilde{x}_{i}' \in \frac{\fancyscript{b}_{i} - \sum _{j = 1}^{i-1} \mathcal {A}_{ij}\fancyscript{x}_{j}' - \sum _{j = i+1}^{n} \mathcal {A}_{ij}\fancyscript{x}_{j} }{\mathcal {A}_{ii}}. \end{aligned}$$

Note that the right side of (21) truncated to $\fancyscript{x}$ can be written in form of the Gauss–Seidel operator $\varGamma$. Denote by $\fancyscript{y}_{i}$ the improved interval union enclosure obtained from $\fancyscript{x}_{i}$ and let

22 $$\begin{aligned} \fancyscript{y}_{i} := \varGamma \left( \mathcal {A}_{ii}, \fancyscript{b}_{i} - \sum _{j = 1}^{i -1}\mathcal {A}_{ij}\fancyscript{y}_{j} - \sum _{j = i + 1}^{n}\mathcal {A}_{ij}\fancyscript{x}_{j}, \fancyscript{x}_{i} \right) . \end{aligned}$$

Finally, we denote by $\varGamma (\mathcal {A}, \fancyscript{b}, \fancyscript{x})$ the Cartesian product of variables $\fancyscript{y}_{1}, \ldots , \fancyscript{y}_{n}$ and we have the following result

#### Proposition 6

Let $\mathcal {A}\in {\mathcal {U}}^{n \times n}$, $\fancyscript{b}\in {\mathcal {U}}^{n}$ and $\fancyscript{x}\in {\mathcal {U}}^{n}$. Then

23 $$\begin{aligned} \mathcal {A}' \subseteq \mathcal {A},~ \fancyscript{b}' \subseteq \fancyscript{b},~ \fancyscript{x}' \subseteq \fancyscript{x}&~~~\Rightarrow ~~~&\varGamma (\mathcal {A}', \fancyscript{b}', \fancyscript{x}') \subseteq \varGamma (\mathcal {A}, \fancyscript{b}, \fancyscript{x}). \end{aligned}$$

24 $$\begin{aligned} \tilde{x} \in \varSigma (\mathcal {A}, \fancyscript{b})\cap \fancyscript{x}&~~~\Rightarrow ~~~&\tilde{x} \in \varGamma (\mathcal {A}, \fancyscript{b}, \fancyscript{x}), \end{aligned}$$

#### Proof

Relation (23) follows from the component-wise application of (20). Since $\tilde{x} \in \varSigma (\mathcal {A}, \fancyscript{b})\cap \fancyscript{x}$, there are $A \in \mathcal {A}$ and $b \in \fancyscript{b}$ such that $A \tilde{x} = b$. Relation (24) follows from (21) and Definition (22). $\square$

### Partial form

We implement the partial Gauss–Seidel procedure that is based on the Gauss–Seidel operator (15) in Algorithm 1. We incorporate Relations (18) and (19) to the algorithm in order to avoid unnecessary divisions. We stop the algorithm when the following criteria are reached for $\epsilon _{Abs} > 0$ and $\epsilon _{Rel} > 0$

25 $$\begin{aligned} \max \hbox {wid}(\fancyscript{x}) - \max \hbox {wid}(\fancyscript{y})< \epsilon _{Abs}~~~ \text{ and } ~~~ 1 - \frac{\max \hbox {wid}(\fancyscript{y})}{\max \hbox {wid}(\fancyscript{x})} < \epsilon _{Rel}. \end{aligned}$$

**Fig. 1:**
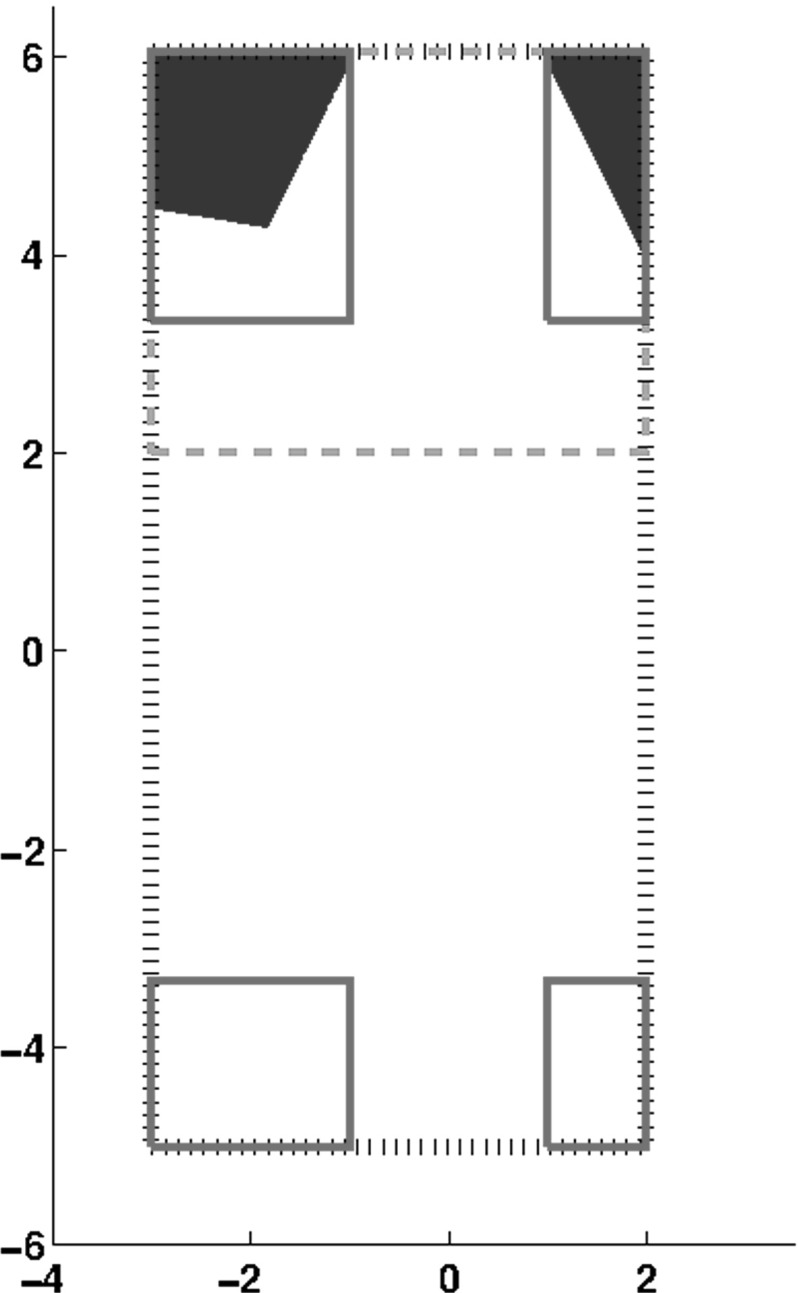
Solution set of Example 1 and enclosures obtained with the partial form of the Gauss–Seidel procedure. The initial box is given in the *outer dotted line*. The enclosure obtained with one iteration of the interval Gauss–Seidel is given by the *dashed box* (an improvement of $63\%$ in volume and $54\%$ in the maximum width w.r.t the the initial box). The enclosures obtained by the interval union Gauss–Seidel procedure with $K = 1$ are given in *solid lines* (an improvement of $76\%$ in volume and $60\%$ in the maximum width w.r.t the initial box)


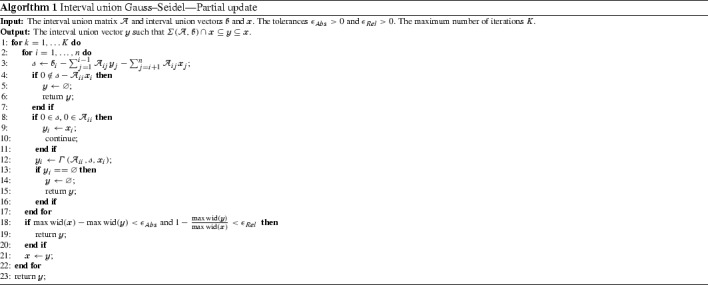


#### Example 1

Let $\mathcal {A}$, $\fancyscript{b}$ and $\fancyscript{x}$ be given by

$$\begin{aligned} \mathcal {A}= \left( \begin{array}{c@{\quad }c} \{[-2.0, 2.0]\}&{} \{[0.5, 1.0]\} \\ \{[0.5, 1.0]\} &{} \{ [-3.0, 3.0]\}\\ \end{array} \right) ,~~~ \fancyscript{b}= \left( \begin{array}{c} \{[8.0, 8.0]\} \\ \{[12.0, 12.0]\} \\ \end{array} \right) \end{aligned}$$

and $\fancyscript{x}= (\{[-3, 2]\}, \{[-5, 6]\})^{T}$. The solution set $\varSigma (\mathcal {A}, \fancyscript{b})\cap \fancyscript{x}$ for this problem as well as the enclosures obtained by interval and interval union algorithms are shown in Fig. 1. Since $\mathcal {A}_{ij}, \fancyscript{b}_{i}, \fancyscript{x}_{i} \in {\mathcal {U}}_{1}$ for every *i* and *j*, we can compare the performance of Algorithm 1 with the traditional interval Gauss–Seidel procedure.

The interval Gauss–Seidel procedure applied to the permuted matrix gives

$$\begin{aligned} \mathbf{x}_{I} = ([-3, 2], [2, 6])^{T}. \end{aligned}$$

This is an improvement of $63\%$ in volume and $54\%$ in the maximum width compared to the initial box. We describe now the application of Algorithm 1 to the problem. In this case, the interval union Gauss–Seidel procedure solves the problem directly, without any permutation.

In the first iteration $(i = 1)$ we have

$$\begin{aligned} \fancyscript{s}= \left\{ [8.0, 8.0]\} - \{[0.5, 1.0]\} \{[-5, 6]\} = \{[2.0, 13.0] \right\} . \end{aligned}$$

Since $\mathcal {A}_{11}\fancyscript{x}_{1} = \{[-6.0, 6.0]\}$ follows that $0 \in \fancyscript{s}- \mathcal {A}_{11}\fancyscript{x}_{1}$ and $0 \notin \fancyscript{s}$. Applying the Gauss–Seidel operator we obtain

$$\begin{aligned} \fancyscript{y}_{1} = \left\{ [-3, -1], [1, 2] \right\} \end{aligned}$$

and conclude the first iteration. The second iteration ($i = 2$) starts with

$$\begin{aligned} \fancyscript{s}= \left\{ [10, 11.5], [12.5, 15] \right\} . \end{aligned}$$

In this case, $\mathcal {A}_{2}\fancyscript{x}_{2} = \{[-18, 18]\}$ and applying the Gauss–Seidel operator we have

$$\begin{aligned} \fancyscript{y}_{2} = \left\{ [-5, -3.3333], [3.3333, 6] \right\} \end{aligned}$$

and we finish the internal loop. The interval union Gauss–Seidel procedure produces 4 disjoint boxes representing an improvement of $76\%$ in volume and $60\%$ in maximum width compared to the initial box. There is no improvement in $\fancyscript{y}_{1}$ and $\fancyscript{y}_{2}$ if we set $K = 2$ in Algorithm 1.

### Complete form

Algorithm 1 is said to be partial since it considers only the variable corresponding to the diagonal entry at each iteration. In the following, we present the complete Gauss–Seidel procedure. It applies the Gauss–Seidel operator to all variables at each iteration.

The solution set obtained by the complete Gauss–Seidel procedure is at least as good as those given by the partial version. On the other hand, the complete procedure requires more calculations and may be prohibitive in higher dimensions.

In order to improve the efficiency of the complete Gauss–Seidel, we apply inner subtraction to each row. Note that the Gauss–Seidel operator applied to the variable $\fancyscript{x}_{j}$ in the *i*th row is given by

$$\begin{aligned} \varGamma \left( \mathcal {A}_{ij}, \fancyscript{b}_{i} - \sum _{\begin{array}{c} k = 1 \\ k \ne j \end{array}}^{n} \mathcal {A}_{ik} \fancyscript{x}_{k}, \fancyscript{x}_{j} \right) . \end{aligned}$$

Considering the auxiliary variable $\fancyscript{s}:= \fancyscript{b}_{i} - \sum _{k = 1}^{n} \mathcal {A}_{ik} \fancyscript{x}_{k}$, the Gauss–Seidel operation becomes

$$\begin{aligned} \varGamma \left( \mathcal {A}_{ij}, \fancyscript{s}\ominus \mathbf{A}_{ij}\fancyscript{x}_{j}, \fancyscript{x}_{j} \right) \end{aligned}$$

where $\ominus$ is interval union generalization of the inner subtraction defined by Equation (3). Algorithm 2 gives the complete form of the interval union Gauss–Seidel procedure. It also implements Relations (18) and (19) to avoid unnecessary divisions. The stopping criteria adopted to this algorithm are the same as in the Algorithm 1.
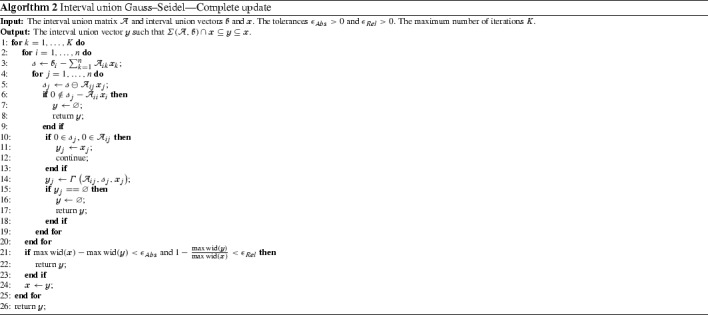


#### Example 2

**Fig. 2:**
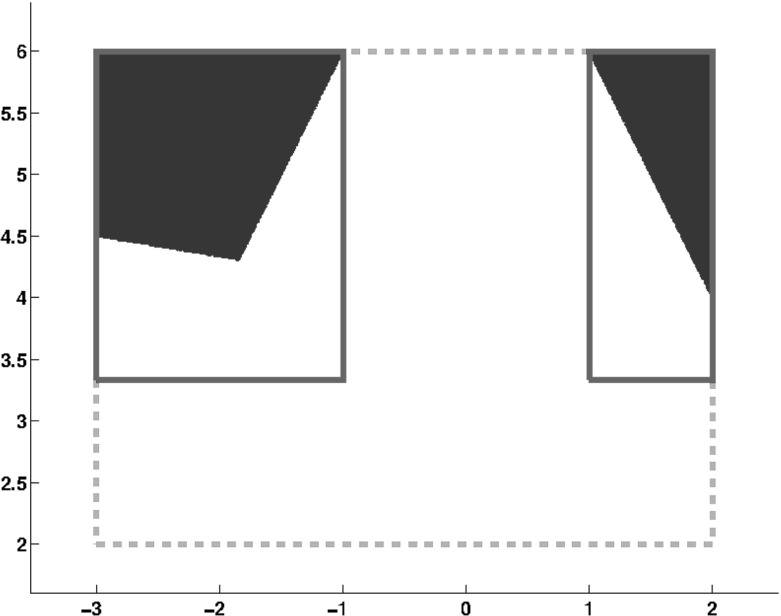
Solution set of Example 1 with complete interval union Gauss–Seidel. The solution obtained with one iteration of the interval Gauss–Seidel is given by the *dashed box*. The solution obtained by the interval union Gauss–Seidel with $K = 1$ is given in *solid lines*

(Example 1 revisited) Let $\mathcal {A}$, $\fancyscript{b}$ and $\fancyscript{x}$ be given as in Example 1. The solution sets obtained by the application of the complete form of the interval and interval union Gauss–Seidel procedures are given in Fig. 2.

The complete interval Gauss–Seidel procedure produces the same result as the partial form and therefore $\mathbf{x}_{I} = ([-3, 2], [2,6])^{T}$.

Applying the complete form with interval unions we obtain

$$\begin{aligned} \fancyscript{y}_{1} = \left\{ [-3, -1], [3.3333, 6]\right\} \text { and } \fancyscript{y}_{2} = \left\{ [1, 2], [3.3333, 6]\right\} \end{aligned}$$

representing an improvement of $85\%$ in volume and $72\%$ in the maximum width compared to the initial box. Note that the complete form removes two interval boxes that do not contain any solution and that could not be deleted with the partial form (see Figs. 1 and 2). Again, there is no improvement in $\fancyscript{y}_{1}$ and $\fancyscript{y}_{2}$ if we set $K = 2$ in Algorithm 2.

### Gap filling

The number of boxes produced by Algorithms 1 and 2 may increase exponentially by the number of divisions with intervals containing zero. A similar phenomenon was already observed by Hyvönen [11] for the propagation of discontinuous intervals; however, the remedy proposed there—simply to take the interval hull—unnecessarily discards useful information. As a more flexible remedy, [24] introduced the notion of gap filling. In this section we describe a gap filling strategy that (among several strategies tried) proved useful for the interval union Gauss–Seidel procedure.

A gap filling is a mapping $\mathcal {g}: {\mathcal {U}}_{k} \rightarrow {\mathcal {U}}_{k}$ satisfying $\fancyscript{x}\subseteq \mathcal {g}(\fancyscript{x})$ and  for any $\fancyscript{x}\in {\mathcal {U}}_{k}$. Two possible, trivial gap filling would be $\mathcal {g}(\fancyscript{x}) = \fancyscript{x}$ and . The gap filling $\mathcal {g}(\fancyscript{x}) = \fancyscript{x}$ however does not avoid the exponential increase on the number of boxes produced by Algorithms 1 and 2. In contrary, the gap filling  do not lead an increased number of boxes, but also loses valuable gap information. Therefore in Algorithm 3 we propose a gap filling that controls the maximum number of gaps produced.
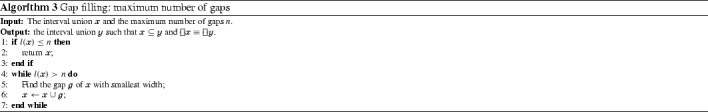


Algorithm 3 can be modified to also handle interval union vectors and matrices. In this case we look for the gap with the smallest width in the whole vector or matrix and fill it in the while loop (Lines 4–7) of the algorithm. Note that using a multi-map data structure in the implementation of the gap filling for vectors and matrices allows faster access to the smallest gaps, improving the overall speed of the algorithm.

## Preconditioners

In this section we present the midpoint and Gauss–Jordan preconditioners for interval union linear systems. It is usually necessary to precondition interval union linear systems of equations to obtain meaningful bounds on the solution set. A preconditioner is any real non-singular matrix *C*.

Given $\mathcal {A}\in {\mathcal {U}}^{n \times n}$, $\fancyscript{b}\in {\mathcal {U}}^{n}$ and $\fancyscript{x}_{0} \in {\mathcal {U}}^{n}$, we are interested in preconditioners satisfying

$$\begin{aligned} \varSigma (\mathcal {A}, \fancyscript{b})\cap \fancyscript{x}_{0} \subseteq \varGamma (C\mathcal {A}, C\fancyscript{b}, \fancyscript{x}_{0}) \subseteq \varGamma (\mathcal {A}, \fancyscript{b}, \fancyscript{x}_{0}). \end{aligned}$$

Since any non-singular matrix can be chosen as preconditioner, there are several heuristics to determine *C* according to the application. In the interval case, the midpoint preconditioner is the common choice in a number of problems. Optimal linear programming preconditioners are designed by [14] in the context of the interval Newton operator and the Gauss–Jordan preconditioner is proposed by [6]. See also [10] and [15] for recent methods on optimal preconditioning.

The midpoint preconditioner in the interval union framework takes the formwhere the midpoint and $\hbox {proj}$ operators are applied component-wise.

The Gauss–Jordan preconditioner is based on the real Gauss–Jordan elimination algorithm with pivot search. Given a square matrix $A \in \mathbb {R}^{n \times n}$, the algorithm computes *C* and a permutation matrix $P \in \mathbb {R}^{n \times n}$ such that

$$\begin{aligned} CAP = I. \end{aligned}$$

In this paper we take . It is worth to note that due to the permutation matrix we apply the Gauss–Seidel procedure to the modified problem

26 $$\begin{aligned} M y = r ~~~ (M \in C \mathcal {A}P, r \in C\fancyscript{b}, y \in \fancyscript{x}_{0}P). \end{aligned}$$

### Example 3

Let $\mathcal {A}$, $\fancyscript{b}$ and $\fancyscript{x}$ be given by

$$\begin{aligned} \mathcal {A}= \left( \begin{array}{cc} \{[0.00, 0.14]\}&{} \quad \{[0.54, 1.23]\} \\ \{[-0.06, 1.67]\} &{} \quad \{ [0.31, 1.02]\}\\ \end{array} \right) , ~~~ \fancyscript{b}= \left( \begin{array}{c} \{[1.73, 1.73]\} \\ \{[6.76, 6.76]\} \\ \end{array} \right) \end{aligned}$$

and $\fancyscript{x}= (\{[2.5, 3.5]\}, \{[3.0, 4.0]\})^{T}$. Applying the partial form of the Gauss–Seidel operator to each variable without preconditioner gives

$$\begin{aligned} \fancyscript{y}_{1} = \left\{ [2.5, 3.5]\right\} \cap {\mathcal {U}}\left( \frac{[-3.19, 0.11]}{[0, 0.14]}\right) = \left\{ [2.5, 3.5] \right\} \end{aligned}$$

and

$$\begin{aligned} \fancyscript{y}_{2} = \{[3.0, 4.0]\} \cap {\mathcal {U}}\left( \frac{[0.9150, 6.9701]}{[0.31, 1.02]}\right) = \{[3.0, 4.0]\} \end{aligned}$$

On the other hand, the Gauss–Jordan preconditioner presented in this section gives

$$\begin{aligned} C = \left( \begin{array}{cc} 1.20894 &{}\quad -0.10512 \\ -0.99869 &{}\quad 1.32908\\ \end{array} \right) , ~~~ P = \left( \begin{array}{cc} 0 &{}\quad 1\\ 1 &{}\quad 0\\ \end{array} \right) . \end{aligned}$$

The permuted system is given by

$$\begin{aligned} \mathcal {M}= \left( \begin{array}{cc} \{[0.545,1.454]\}&{}\quad \{[-0.175,0.175]\} \\ \{[-0.816,0.816]\} &{}\quad \{[-0.219,2.219]\}\\ \end{array} \right) ,~~~ \fancyscript{r}= \left( \begin{array}{c} \{[1.380,1.380]\} \\ \{[7.256,7.256]\} \\ \end{array} \right) \end{aligned}$$

and $\fancyscript{x}' = (\{[3.0, 4.0]\}, \{[2.5, 3.5]\})^{T}$. We obtain the following bounds with the Gauss–Seidel procedure applied to the permuted system

$$\begin{aligned} \fancyscript{y}_{1}' = \{[3.0, 4.0]\} \cap {\mathcal {U}}\left( \frac{[0.7663, 1.9953]}{[0.5455, 1.4545]}\right) = \{[3.0, 3.65]\} \end{aligned}$$

and

$$\begin{aligned} \fancyscript{y}_{2}' = \{[2.5, 3.5]\} \cap {\mathcal {U}}\left( \frac{[4.2712, 10.2425]}{[-0.2196, 2.2196]}\right) = \{[2.5, 3.5]\}. \end{aligned}$$

The new enclosure represents an improvement of $34\%$ in volume compared to the initial box. Note that we must apply the inverse permute to $\fancyscript{y}_{1}'$ and $\fancyscript{y}_{2}'$ in order to obtain the correct enclosure. In this example, the same result would be obtained by applying the midpoint preconditioner.

The matrix *C* is dense in general. Therefore, preconditioner strategies may be prohibitive in large linear systems of equations. Moreover, systems of form (26) may overestimate the solution set in some problems. For example, let $\mathcal {A}$, $\fancyscript{b}$ and $\fancyscript{x}$ be given by

$$\begin{aligned} \mathcal {A}= \left( \begin{array}{cc} \{[-2.0, 2.0]\}&{}\quad \{[0.5, 1.0]\} \\ \{[0.5, 1.0]\} &{}\quad \{ [2.0, 3.0]\}\\ \end{array} \right) , ~~~ \fancyscript{b}= \left( \begin{array}{c} \{[6.0, 6.0]\} \\ \{[6.0, 6.0]\} \\ \end{array} \right) \end{aligned}$$

and $\fancyscript{x}= (\{[-3, 2]\}, \{[-6, 6]\})^{T}$. If we apply the Algorithm 1 with $\epsilon _{Abs} = \epsilon _{Rel} = 10^{-4}$ and $K = 1$ to the original system, we obtain $\fancyscript{y}_{\text {UNP}} = (\{[-3, 2]\}, \{ [1.333, 4.5]\})^{T}$. The resulting interval union vector represents an improvement of $73\%$ in volume when compared with the initial box. On the other hand, applying the Algorithm 1 with the same parameters to the corresponding system of form (26), obtained with the Gauss–Jordan preconditioning gives $\fancyscript{y}_{\text {GJ}} = (\{[-3, -0.7826], [0.9729, 2]\}, \{ [0, 6]\})^{T}$. The solution vector $\fancyscript{y}_{\text {GJ}}$ is an improvement of $67\%$ in volume when compared with the initial box.

We introduce a mixed strategy that combines the original linear system with its preconditioned form. Given $\mathcal {A}\in {\mathcal {U}}^{n \times n}$, $\fancyscript{b}\in {\mathcal {U}}^{n}$ and $\fancyscript{x}\in {\mathcal {U}}^{n}$ we alternate between the solution of the original system and the preconditioned form (26) until one of the following: (1) we prove that there is no solution in $\fancyscript{x}$, (2) the maximum number of iterations is reached, or (3) we have not enough gain in the last solution of both the original and preconditioned systems.

Algorithm 4 implements the mixed strategy using the partial or complete forms of the interval union Gauss–Seidel procedure. The boolean variables gainUnprec and gainGS control the next iteration of the algorithm. If both are false then neither the Gauss–Seidel procedure without preconditioning nor the same procedure with preconditioning gave a substantial improvement on the current box and the mixed algorithms stops. Algorithm 4 can be modified to apply the midpoint preconditioner instead of the Gauss–Jordan method.
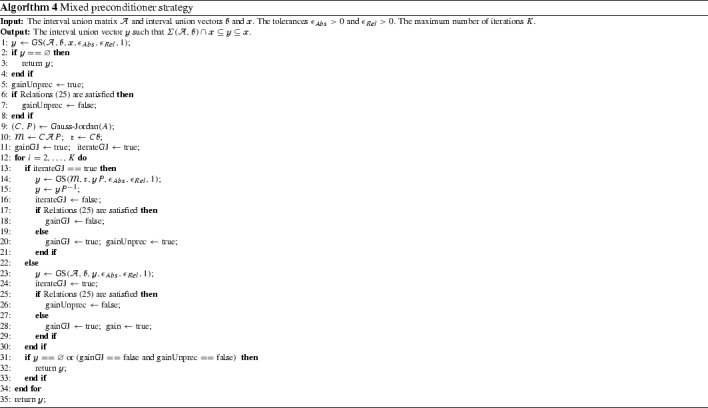


## Numerical experiments

In this section we perform numerical experiments to compare the interval union Gauss–Seidel procedure with its interval counterpart. We consider the partial and complete forms of the Gauss–Seidel procedure as well as the midpoint and the Gauss–Jordan preconditioners. In this test, we take only interval linear systems of equations into account. The experiment is described in Algorithm 5.
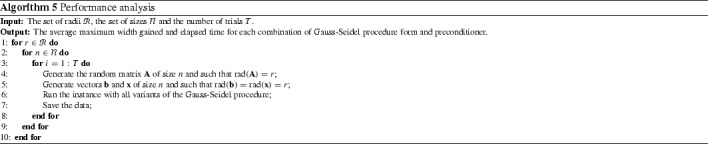


In this section, we set the parameters of the Algorithms 1 and 2 as $\epsilon _{Abs} = \epsilon _{Rel} = 10^{-4}$ and $K = 2$ for the partial form and $K = 1$ for the complete form. In the gap filling Algorithm 3, we set the maximum number of gaps in an interval union as $g = 2$ and the maximum number of boxes for interval union vectors to 64.

In Algorithm 5, we set $\mathcal {R} := \{0.1, 0.2,\ldots , 2.9, 3.0\}$, $\mathcal {N} := \{2, 3, 5, 10, 15, 20, 30, 50\}$ and $T = 100$. The entries of $\mathbf{A}$, $\mathbf{b}$ and $\mathbf{x}$ have radius given by $r \in \mathcal {R}$ and satisfy the rules described in Table 1.

**Table 1:** Description of the processes that generate matrices and vectors $\mathbf{A}$, $\mathbf{b}$ and $\mathbf{x}$

Case	$\mathbf{A}$	$\mathbf{b}$	$\mathbf{x}$
1	$\check{\mathbf{A}}_{ii} \in [-1, 1]$, $\check{\mathbf{A}}_{ij} \in [-5, 5]$	$\check{\mathbf{b}} \in [-1, 1]$	$\check{\mathbf{A}}^{-1}\check{\mathbf{b}} \in \mathbf{x}$
2	$\check{\mathbf{A}}_{ii} \in [-5, 5]$, $\check{\mathbf{A}}_{ij} \in [-1, 1]$	$\check{\mathbf{b}} \in [-1, 1]$	$\check{\mathbf{A}}^{-1}\check{\mathbf{b}} \in \mathbf{x}$
3	$\check{\mathbf{A}}_{ij} \in [-1, 1]$	$\check{\mathbf{b}} \in [-1, 1]$	$\check{\mathbf{x}} \in [-1, 1]$
4	$\check{\mathbf{A}}_{ij} \in [-1, 1]$	$\check{\mathbf{b}} \in [n, 10n]$	$\check{\mathbf{x}} \in [-1, 1]$
5	$\check{\mathbf{A}}_{ii} \in [-1, 1]$, $\check{\mathbf{A}}_{ij} \in [-5, 5]$	$\check{\mathbf{b}} \in [n, 10n]$	$\check{\mathbf{x}} \in [-1, 1]$

The number *n* stands for the dimension of the linear system

**Fig. 3:**
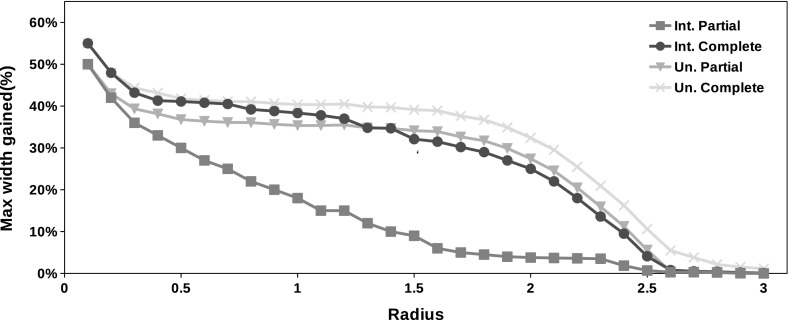
Average maximum width gained with each method in problems of size $n \in \{2, 5, 10, 15, 20, 30, 50\}$. All possible forms of the Gauss–Seidel procedure without preconditioning

**Fig. 4:**
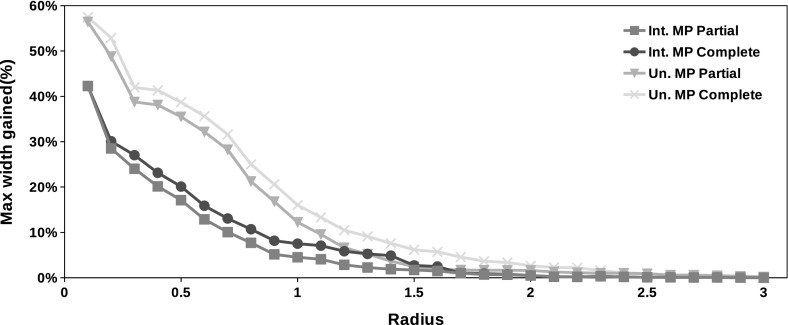
Average maximum width gained with each method in problems of size $n \in \{2, 5, 10, 15, 20, 30, 50\}$. All possible forms of the Gauss–Seidel procedure with the midpoint preconditioner

**Fig. 5:**
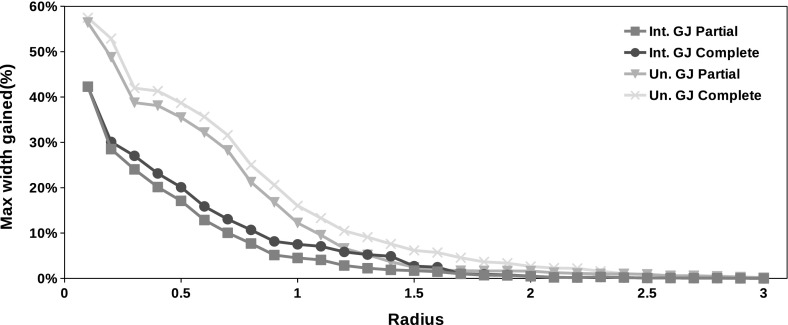
Average maximum width gained with each method in problems of size $n \in \{2, 5, 10, 15, 20, 30, 50\}$. All possible forms of the Gauss–Seidel procedure with the Gauss–Jordan preconditioner

Figures 3, 4 and 5 summarize the results of the experiment. For each point in these graphs we have the average of the maximum width gained with the methods in a set of 4000 problems taken at random (100 for each $n \in \mathcal {N}$ and for each one of the 5 cases displayed in Table 1). Tables 2 and 3 show the average elapsed time for each method. All the algorithms were implemented in *JGloptlab* [4], a Java implementation of the state of the art global optimization algorithms. We run the experiment in a *corei7* processor with 6 Gb of RAM memory.

It is clear that the interval union Gauss–Seidel procedure produces better enclosures than the interval method. Tables 2 and 3 show that there are no significant differences between the execution time of the Gauss–Seidel procedure with intervals and interval unions.

**Fig. 6:**
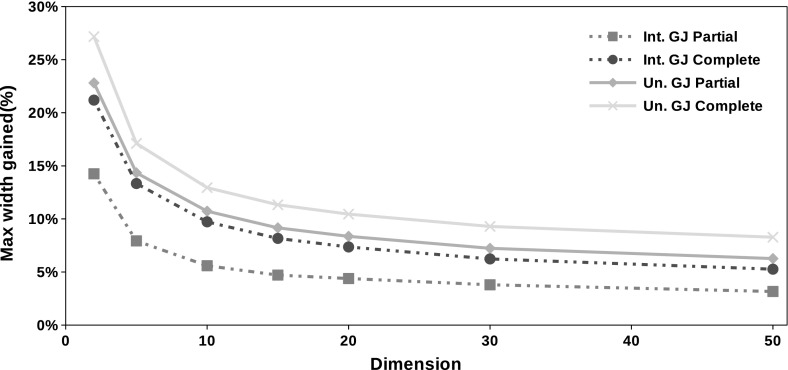
Average maximum width gained with each method in the same problems used on Fig. 5 as function of the dimension

Figure 6 show the effect of the dimension on the quality of the computed enclosures considering the Gauss–Jordan preconditioner.

**Table 2:** Average elapsed time (in seconds) for the partial form

n	Interval			Union		
	Unprec.	Midpoint	Gauss–Jordan	Unprec.	Midpoint	Gauss–Jordan
15	0.001	0.001	0.001	0.001	0.03	0.012
20	0.001	0.39	0.404	0.001	0.538	0.515
30	0.001	3.135	3.23	0.001	3.621	3.726
50	0.001	15.806	16.752	0.001	16.72	17.772

*Unprec.* stands for algorithms without preconditioning

**Table 3:** Average elapsed time (in seconds) for the complete form

n	Interval			Union		
	Unprec.	Midpoint	Gauss–Jordan	Unprec.	Midpoint	Gauss–Jordan
15	0.001	0.003	0.001	0.001	0.041	0.043
20	0.001	0.396	0.408	0.001	0.696	0.655
30	0.009	3.163	3.266	0.001	3.812	3.898
50	0.004	15.986	16.89	0.001	17.689	18.644

*Unprec.* stands for algorithms without preconditioning

**Fig. 7:**
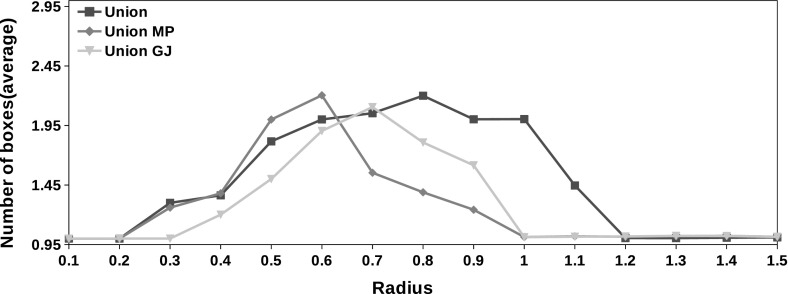
Maximum number of boxes generated during the execution of the partial form of the interval union Gauss–Seidel procedure in average. $\textit{MP}$ stands for the midpoint precoditioner and $\textit{GJ}$ denotes the method with the Gauss–Jordan preconditioner

**Fig. 8:**
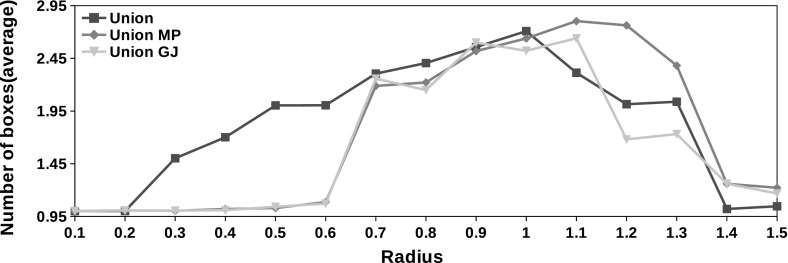
Maximum number of boxes generated during the execution of the complete form of the interval union Gauss–Seidel procedure in average. $\textit{MP}$ stands for the midpoint preconditioner and $\textit{GJ}$ denotes the method with the Gauss–Jordan preconditioner

The exponential increase in the number of boxes produced by Algorithms 1 and 2 is one of the main concerns regarding the use of the interval union arithmetic. We note that the maximum number of boxes produced in during the interval union Gauss–Seidel procedure is, in average, never greater than 3 as showed by Figs. 7 and 8. Moreover, we reach the maximum number of boxes prescribed in Algorithm 3 during the execution of the procedure only in $10\%$ of the 120,000 instances with the complete form. We never reach the maximum number of boxes with the partial form.

**Fig. 9:**
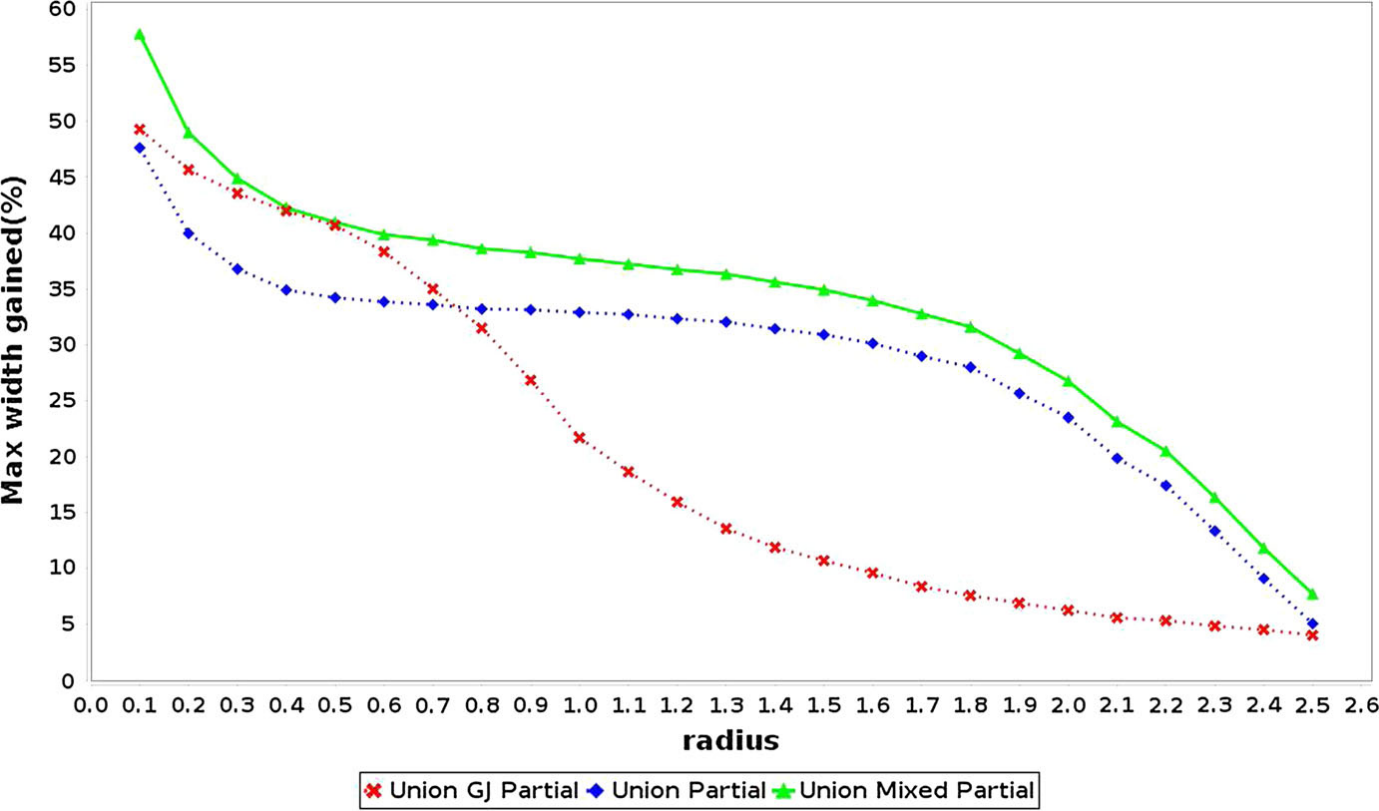
Average maximum width gained with each method in problems of size $n \in \{2, 5, 10, 15, 20, 30, 50\}$. The figure displays the partial form of the interval union Gauss–Seidel without preconditioner, with the Gauss–Jordan preconditioner and the mixed strategy

**Fig. 10:**
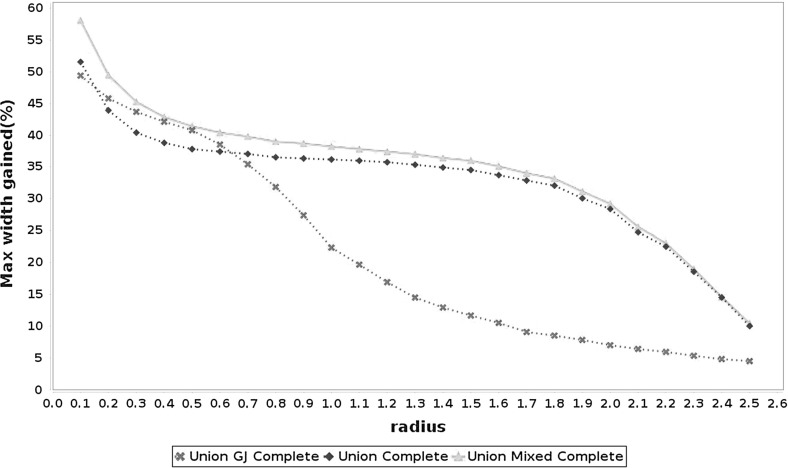
Average maximum width gained with each method in problems of size $n \in \{2, 5, 10, 15, 20, 30, 50\}$. The figure displays the complete form of the interval union Gauss–Seidel without preconditioner, with the Gauss–Jordan preconditioner and the mixed strategy

**Table 4:** Average elapsed time (in seconds) for the partial and complete forms

n	Partial			Complete		
	Unprec.	Gauss–Jordan	Mixed	Unprec.	Gauss–Jordan	Mixed
15	0.015	2.583	0.919	0.121	6.479	2.835
20	0.026	16.582	12.765	1.125	21.56	16.471
30	0.106	59.697	50.741	12.361	73.247	66.726
50	2.9	285.19	252.845	54.105	325.764	314.148

*Unprec.* is the interval union Gauss–Seidel without preconditioner and Mixed is the strategy described in Algorithm 4

### Mixed preconditioner strategy

It is clear from Tables 2 and 3 that the interval union Gauss–Seidel procedure without preconditioner is several times faster than the same method with preconditioners. Moreover, there are problems where the preconditioner leads to poorer bounds than the solution of the original system.

We finish this section comparing Algorithms 1 and 2 with the mixed strategy proposed in Algorithm 4. In this experiment we set the parameters of all algorithms as $\epsilon _{Abs} = \epsilon _{Rel} = 10^{-4}$ and $K = 2$. We perform the experiment in the same test set described previously.

Figures 9 and 10 show the results of the experiment. Table 4 compares the average elapsed time for each method.

The figures show that the mixed strategy produces bounds that are, in average, sharper than those obtained with simple methods. It can be explained by the observation that there is no dominant preconditioner strategy. The Gauss–Jordan preconditioner is better suited to cope with some problems (for example, ill conditioned problems) while the original system provides better solutions in other classes of interval linear systems (for example, diagonally dominant). On the other hand, Table 4 shows that the mixed strategy is not faster than the Gauss–Jordan preconditioner. It is due to the fact that in many problems the second iteration of the Algorithm 4 is needed.

## Concluding remarks

In this paper, we introduce the interval union Gauss–Seidel procedure to rigorously enclose the solution set of

$$\begin{aligned} A x = b ~~~ (A \in \mathcal {A}, b \in \fancyscript{b}, x \in \fancyscript{x}_{0}). \end{aligned}$$

The Gauss–Seidel procedure is presented in two forms; the partial one (Algorithm 1) and the complete one (Algorithm 2). At each iteration, in the former we update only the variable corresponding to the main diagonal of the matrix $\mathcal {A}$, whereas in the latter every variable is updated.

We also studied two preconditioner heuristics for the interval union Gauss–Seidel procedure. The midpoint preconditioner takes the inverse of the midpoint of the interval hull of $\mathcal {A}$ and the Gauss–Jordan preconditioner that is based on the interval version of this method discussed by [6]. We also propose a mixed strategy that combines the original system and the Gauss–Jordan preconditioner to improve the efficiency and the quality of solutions, see the Algorithm 4.

Numerical experiments show that the interval union Gauss–Seidel procedure produces better enclosures than its interval counterparts. We performed tests on 120,000 problems generated at random as described by Table 1. Figures 3, 4 and 5 demonstrate that interval union procedures produce bounds that are up to $25\%$ sharper than those obtained by the interval implementation of the method. Tables 2 and 3 show that there is no disadvantage in computation time when using interval union methods as compared to interval ones.

The potential increase in the number of boxes produced by Algorithms 1 and 2 is one of the main concerns in the use of interval union methods. We propose a gap filling strategy based on the ideas described by [24]. The resulting method is given by 3. We show that the maximum number of boxes produced by the complete form of the Gauss–Seidel procedure is reached only in $10\%$ of instances. We never reach the maximum number of boxes with the partial form. The average number of boxes generated in this experiment is given by Figs. 7 and 8.

We note that the mixed strategy described in Algorithm 4 is faster and more accurate than the interval union Gauss–Seidel procedure with Gauss–Jordan preconditioner. It also produces better enclosures than those obtained with the method without preconditioner. On the other hand, if the maximum radius of $\mathcal {A}$, $\fancyscript{b}$ and $\fancyscript{x}$ are small enough then it is more efficient to turn off the preconditioning as suggested by Figs. 9 and 10.
